# 

**Published:** 1846-08

**Authors:** 


### WESTERN JOURNAL

OF

### MEDICINE AND SURGERY.

### EDITORS.

DANIEL DRAKE, M. D.,

PROFESSOR OF PATHOLOGY AND THE PRACTICE OF MEDICINE
IN THE MEDICAL INSTITUTE OF LOUISVILLE:

LUNSFORD P. YANDELL, M. D.,

PROFESSOR OF CHEMISTRY AND PHARMACY IN THE SAME:

THOMAS W. COLESCOTT, M. D.

TO OUR SUBSCRIBERS.—Many of our subscribers, within a year, have paid up like men; to such we return our thanks; but others are shamefully in arrears, and of these we have to say that they are not true to us, to their profession, or themselves. A medical journal, any more than war, cannot be carried on without money. We are expending money freely upon the Western Journal of Medicine, and once more say to delinquent subscribers, remit immediately.

August 1846. PRENTICE & WEISSINGER.

### RECEIPTS OF MEDICAL JOURNAL FOR JULY, 1846.

| | |
|---|---|
| Dr. P. Capshaw, Whitesburg, Ala., | $10 00 |
| T. R. Beck, Albany, N. Y., | 5 00 |
| W. M. Dodson, Erie, Mo., | 5 00 |
| A. J Embree, Pine Bluff, Ark., | 10 00 |
| J. C. Cook, Newport, Ind. | 5 00 |
| S. W. Horn, Roscoe, Mo., | 5 00 |
| E. Daniels, Terre Haute, Ind., | 10 00 |
| J. G. McMakin, Crawfordsville, Ind., | 15 00 |
| Helm, Springfield, Ill., | 10 00 |
| S. Christy, Farmington, Ill., | 15 00 |
| English, Jacksonville, Ill. | 10 00 |
| E. Dreshback, Tiffin, Ohio, | 5 00 |
| H. Kuhn, do. do. | 10 00 |
| J. Q. Ransom, Lower Sandusky, Ohio, | 5 00 |
| C. Smith, Toledo, Ohio, | 5 00 |
| Baker & Kittridge, Norwalk, Ohio, | 5 00 |

